# Anterior cruciate ligament remnant cells have different potentials for cell differentiation based on their location

**DOI:** 10.1038/s41598-020-60047-w

**Published:** 2020-02-20

**Authors:** Jin Kyu Lee, Sungsin Jo, Young Lim Lee, Hyosun Park, Jun-Seob Song, Il-Hoon Sung, Tae-Hwan Kim

**Affiliations:** 1 Department of Orthopaedic Surgery, Hanyang University Hospital, Seoul, Republic of Korea; 2 Hanyang University Institute for Rheumatology Research, Seoul, Republic of Korea; 3 Department of Bioenvironmental Technology, College of Natural Sciences, Seoul Women’s University, Seoul, Republic of Korea; 4 Department of Orthopaedic Surgery, Gangnam JS Hospital, Seoul, Republic of Korea; 5 Department of Rheumatology, Hanyang University Hospital for Rheumatic Diseases, Seoul, Republic of Korea

**Keywords:** Multipotent stem cells, Ligaments, Stem-cell research

## Abstract

Histological and cytological observations of the human anterior cruciate ligament (ACL) had been described, but the differentiation potency based on their location is still unknown. To determine and compare proliferation and differentiation potential of cells derived from distal and middle thirds of the ACL remnant, ACL remnant was initially marked at the distal third (within 10  mm from the tibial insertion) and middle third (between 10–20  mm from the tibial insertion) and then dissected. Both the middle and distal third regions of ACL remnant were analyzed using CD34^+^ cell counting. Cell proliferation rate did not differ in both middle and distal third regions of ACL remnant, but they showed different characteristics in cell differentiation depending on their location. The distal third region of the ACL remnant had a tendency for chondrogenic differentiation with higher expression of CD34^+^ cells. On the other hand, the middle third region of ACL remnant had a strong tendency for osteogenic and ligamentous differentiation. Characteristics of the ACL remnant tissues should be considered when performing remnant-preserving or harvesting ACL remnants for tissue engineering.

## Introduction

Anterior cruciate ligament reconstruction (ACLR) is one of the most common surgical procedures in the field of orthopaedic sports medicine, with more than 130,000 procedures performed annually in the United States alone^1,2^. It has been well documented that a completely ruptured ACL does not spontaneously heal because of poor vascular supply and an unfavourable intra-articular environment^3^. Given the importance of its biomechanical function, surgical treatment is generally accepted as the standard procedure for restoring knee stability. In most cases, non-augmented primary repair has been unsuccessful, and therefore ACL reconstruction is required^1,4,5^.

For surgical success, ACLR requires tendon graft healing in a surgically created bone tunnel and maturation (i.e., ligamentization) of the graft substance^4,6–9^. Indeed, the lack of vascularity within the tendon graft induces degeneration or micro ruptures during the early postoperative period^10^. To overcome these issues, tissue engineering using stem cells has been widely explored as a means to achieve early graft healing, tendon regeneration, and bone integration. Recently, reports have shown that ruptured human ACL tissues can possess numerous vascular-derived stem cells and that ACL-derived CD34^+^ cells can promote healing and have high expansion and multilineage differentiation potential^7,11–13^. Mifune *et al*.^14^ demonstrated that ACL-derived CD34^+^ cells contributed to tendon-bone healing after ACLR via angiogenesis and osteogenesis enhancements^15^. Furthermore, Matsumoto *et al*.^16^ found that incorporation of ruptured ACL tissues in autologous grafts reduced tunnel enlargement in ACLR^16,17^. However, with regard to their clinical application, potential advantages of remnant-derived stem cells are still questionable. Histological observations of the uninjured human ACL have shown a different composition of cells in different ACL regions: chondrocyte-like cells were predominantly found in the more distal area (approximately 10  mm proximal to the tibial ligament insertion), while fibroblasts were predominantly found in the more proximal region (approximately 25  mm proximal of the tibial ligament insertion)^18^. Therefore, with remnant cells having the potential for expansion and multilineage differentiation, ruptured human ACL remnant tissue where the ACL contains numerous vascular-derived stem cells may show different characteristics in cell differentiation by their location. Based on these latter findings, we performed experiments to test the hypothesis that human ACL remnant cells acquired from the site of ACL rupture have different potentials in cell differentiation based on their location.

## Results

### Differential CD34 level in ACL remnant cells

**Figure 1:**
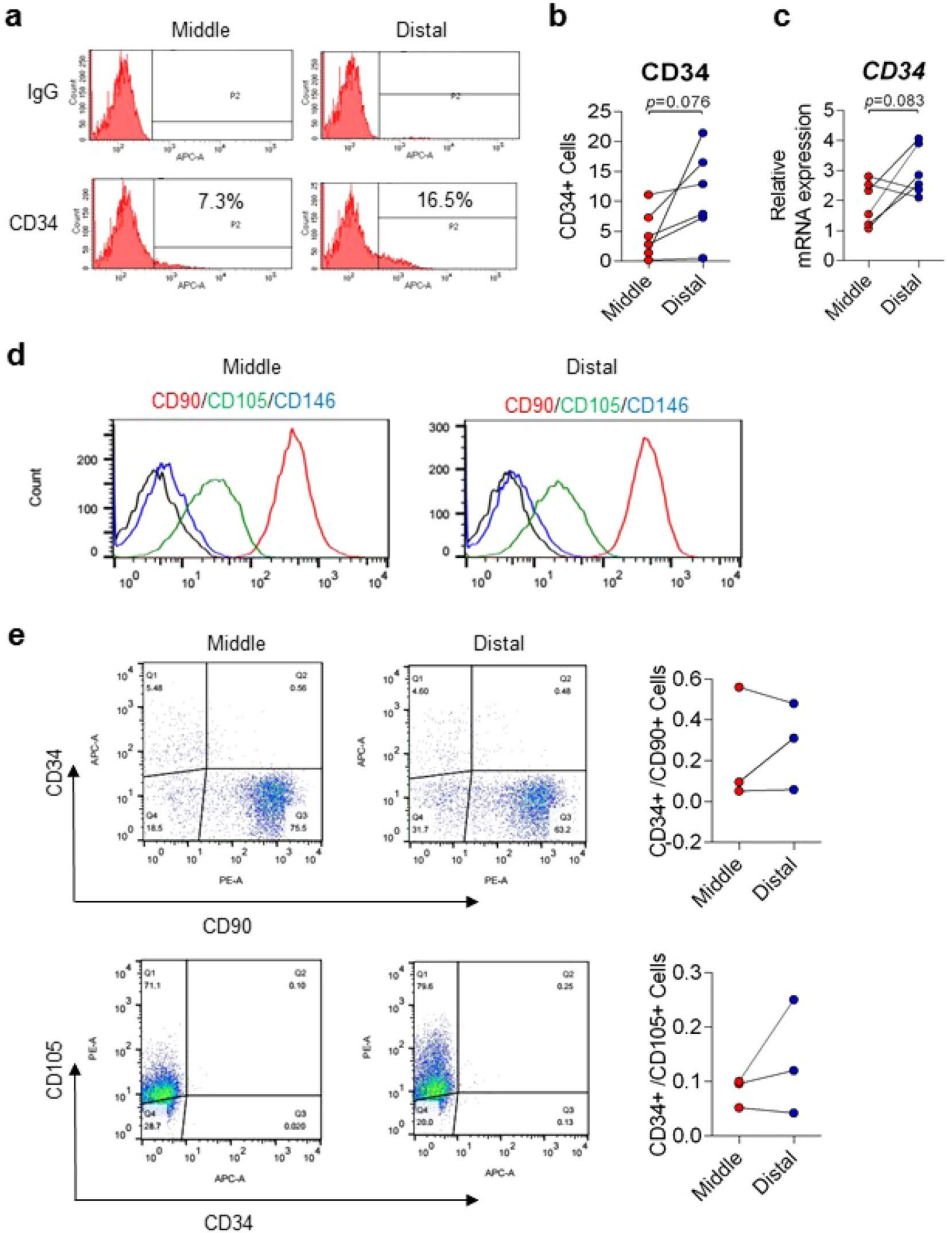
Differential CD34 level in cells derived from middle and distal third of ACL remnant regions. (**a**) CD34 levels in middle and distal cells of ACL remnant regions were analysed by FACS analysis. (**b**) Quantification of (**a**). Red, Middle (n = 6); Blue, Distal (n = 6). (**c**) CD34 mRNA expression was confirmed by qRT-PCR. Red, Middle (n = 6); Blue, Distal (n = 6). Representative FACS data are shown. Error bars show standard error of the mean (n = 6). *p < 0.05. (**d**) CD90, CD105, and CD146 in middle and distal cells of ACL remnant regions was analysed by FACS. Red, CD90; Green, CD105; Blue, CD146. (**e**) Co-expressions of CD34 with CD90 or CD105 were analysed by FACS. Red, Middle (n = 3); Blue, Distal (n = 3).

FACS analysis and qRT-PCR data showed higher population of CD34 positive cells in the distal third region of the ACL remnants than in the middle third region, although it did not reach statistical significance (Fig. 1a,b,c). Histological analysis and immunostaining also displayed that distal third region have many blood vessels and expressing CD34 (Supp. Data 1). Moreover, there were no significant differences of surface markers between middle and distal cells (Fig. 1d) and co-expressions of CD34 with CD90 or CD105 in both (Fig. 1e), but both subpopulations were characterized by the high expression of CD90, moderate expression of CD105, and low expression of CD146. These data indicate that cell distribution in the distal of ACL remnants is more positive for CD34 expression than in the middle.

### Expansion potential and basal characteristics of ACL remnant cells

**Figure 2:**
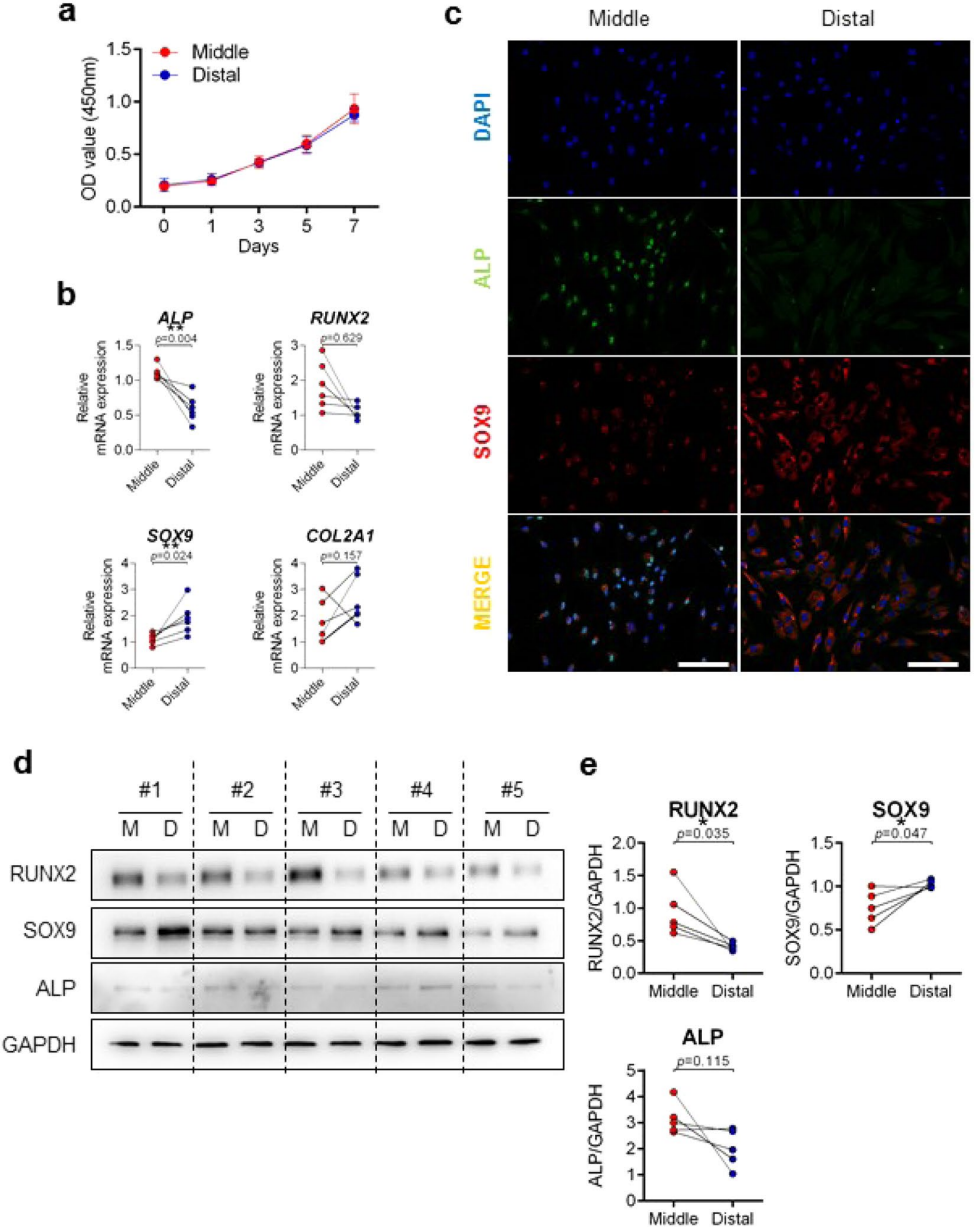
Comparison of basal levels in cells derived from middle and distal third of ACLR regions. (**a**) Cell proliferation rates were determined using a WST assay. X axis: days; Y axis: OD value (450  nm). Red, Middle (n = 5); Blue, Distal (n = 5). (**b**) mRNA expressions of ALP, RUNX2, SOX9, and COL2 were compared by qRT-PCR. Red, Middle (n = 6); Blue, Distal (n = 6). (**c**) Expressions of ALP and SOX9 in both middle and distal cells were compared by immunofluorescence. Green, ALP-alexa-488; Red, SOX9-cy3; Blue, DAPI; Scale bar: 200 μm. Representative image data are shown (n = 3). (**d**) Expression of RUNX2, SOX9, ALP, and GAPDH were detected by immunoblotting (n = 5). (**e**) Quantification of (**d**). Red, Middle (n = 5); Blue, Distal (n = 5). Error bars show standard error of the mean. *p < 0.05. **p < 0.01.

Cell proliferation potential was determined using water-soluble tetrazolium salt (WST), and cell proliferation rates were comparable between the distal and middle third regions during seven days of growth. Cell proliferation rates of both cells did not differ (Fig. 2a). When basal mRNA expression levels were compared, upregulation of ALP and RUNX2 was observed in the middle third region (p = 0.004 and p = 0.629, respectively), while upregulation of SOX9 and collagen type 2 (COL2) was observed in the distal third region (p = 0.024 and p = 0.157, respectively) (Fig. 2b). Immunofluorescence and immunoblotting analysis further confirmed upregulation of ALP and SOX9 in the middle third and distal third regions, respectively (Fig. 2c,d). The immunoblotting results of RUNX2, SOX9, and ALP proteins were quantified and shown as Fig. 2e.

### Multilineage differentiation of ACL remnant cells

#### Osteogenic differentiation

**Figure 3:**
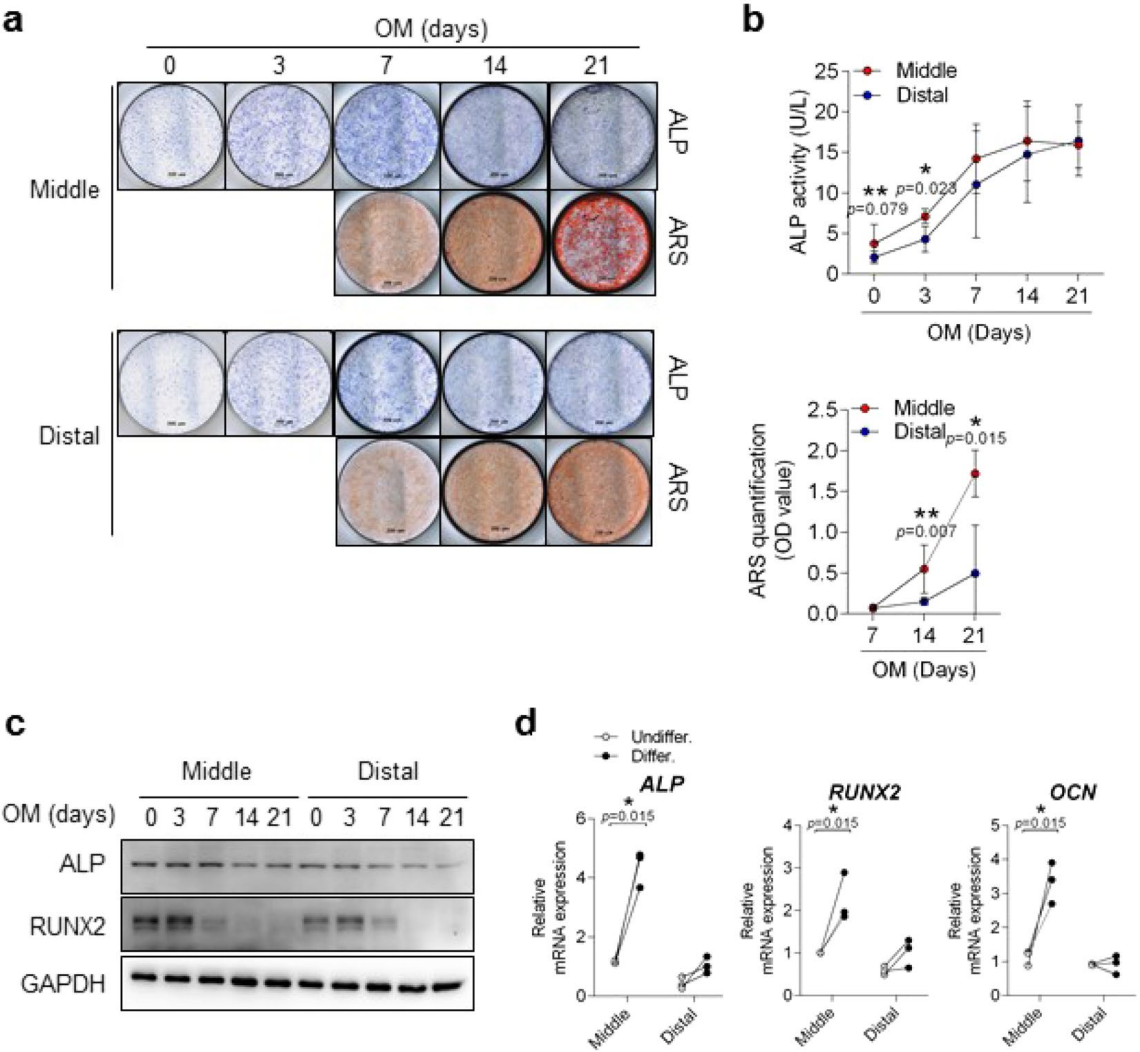
Comparison of osteogenic potential in cells derived from middle and distal third of ACL remnant regions. (**a**) Both ACL remnant cells were induced into osteogenic differentiation as determined by ALP and ARS staining on the indicated days. Representative images are shown. (**b**) Differentiated cells were assessed by intercellular ALP activity (upper) and quantitative ARS staining (lower). Red, Middle (n = 6); Blue, Distal (n = 6). (**c**) Protein expressions in differentiated cells were detected by immunoblotting. Representative images are shown (n = 3). (**d**) mRNA expression of differentiated cells at three days was determined by qRT-PCR. Osteogenic-related genes: ALP, RUNX2, and OCN. Open circle, Undifferentiation (n = 3); Closed circle, Differentiation (n = 3). Error bars show standard error of the mean. *p < 0.05. **p < 0.01.

Remnant cells from the middle third region showed strong staining in ALP and alizarin red (ARS) (Fig. 3). In the ALP activity and ARS quantification assays, the middle third region showed significantly higher ALP expression in the first three days (Fig. 3b, upper) and higher ARS staining after 14 days (up to 21 days) of differentiation (p < 0.05) (Fig. 3b, lower). When comparing osteogenic differentiation, the expression of ALP and RUNX2 proteins was more increased up to 3 days in middle third region and thereafter gradually decreased. In addition, mRNA expressions of ALP, RUNX2, and OCN at 3 days were significantly increased in the middle third region after the osteogenic differentiation stimulus was given (p = 0.015) (Fig. 3d). Notably, the osteogenic differentiation potential was higher in the middle third region of the ACL than in the distal third region.

#### Chondrogenic differentiation

**Figure 4:**
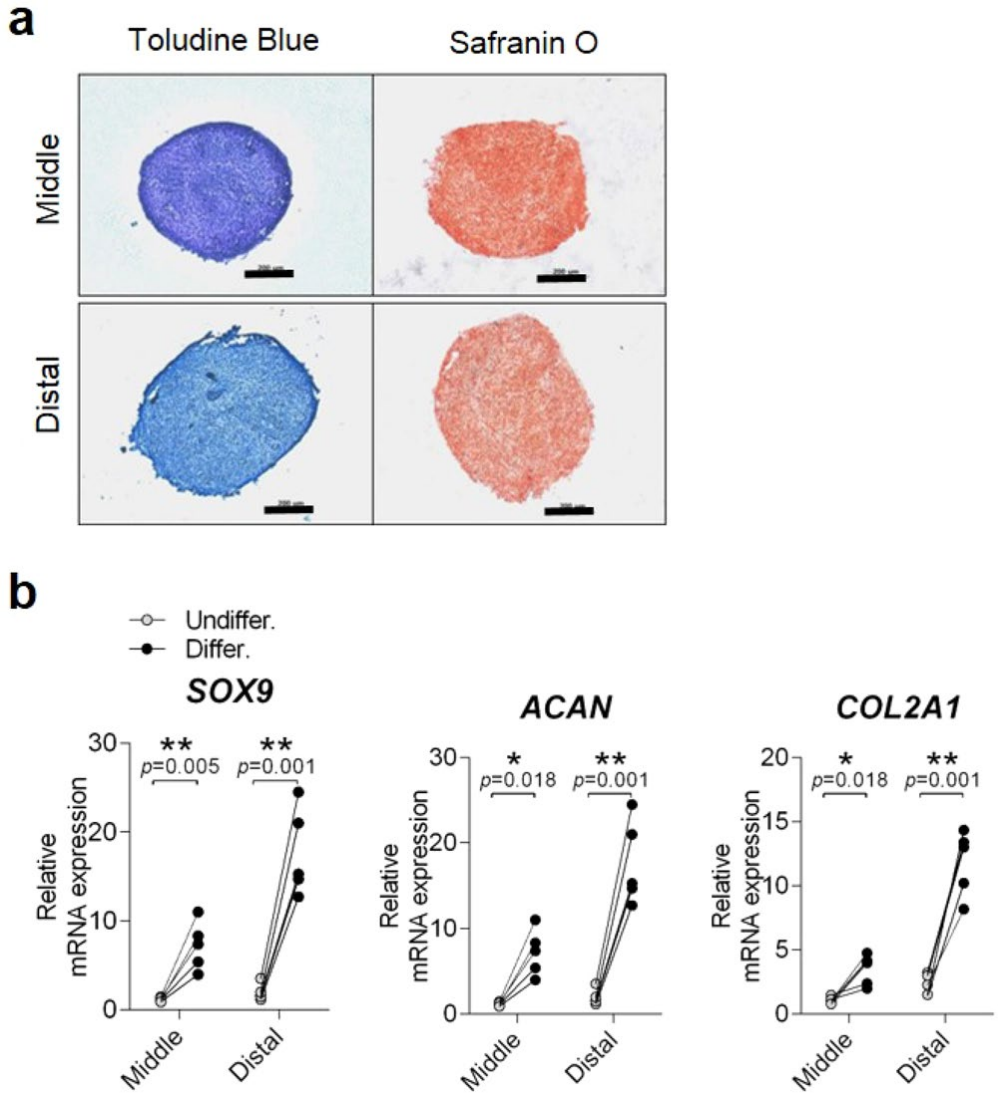
Comparison of chondrogenic potential in cells derived from middle and distal third of ACL remnant regions. (**a**) Both ACL remnant cells were induced into chondrogenic differentiation as determined by toluidine blue and safranin O staining at 35 days. Representative images are shown (n = 5). Scale bar: 200 μm. (**b**) mRNA expression of differentiated cells at 35 days was confirmed by qRT-PCR (n = 5). Chondrogenic-related genes: ACAN, COL2, and SOX9. Open circle, Undifferentiation (n = 5); Closed circle, Differentiation (n = 5). Error bars show standard error of the mean. *p < 0.05. **p < 0.01.

Remnant cells of the distal third region showed better staining by toluidine blue and safranin O than the middle third region (Fig. 4a and Supp. Data 2). Although significant increases of chondrogenic markers (SOX9, ACAN, and COL2) were noted in both regions after the chondrogenic stimulus was given, the increase was greater in the distal third region compared to the middle third region (p = 0.001) (Fig. 4b). Collectively, the chondrogenic differentiation potential was relatively higher in the distal third region than in the middle third region.

#### Ligamentous differentiation

**Figure 5:**
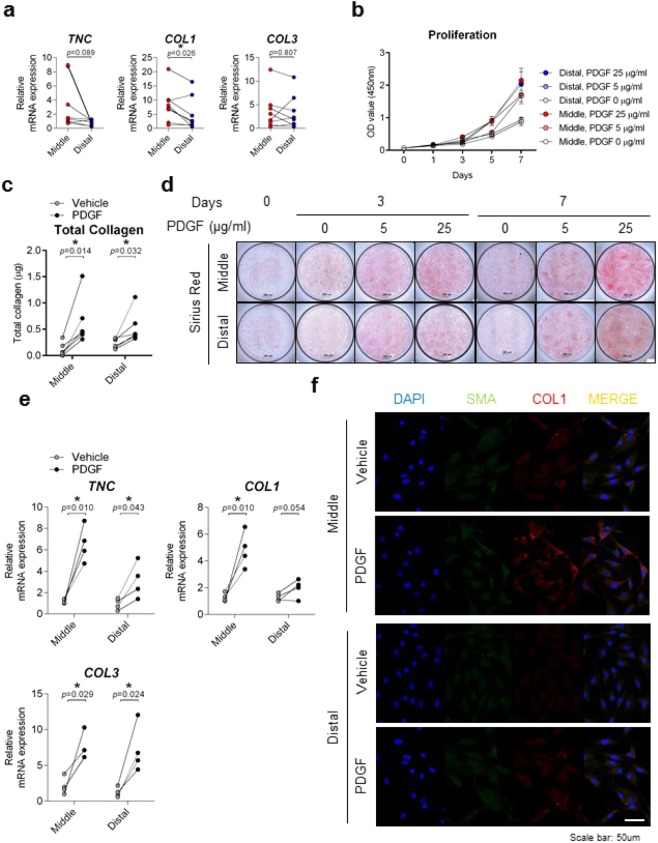
Comparison of ligamentous potential in cells derived from middle and distal third of ACL remnant regions. (**a**) mRNA expressions were compared by qRT-PCR. Ligamentous-related genes: COL1, COL3, and TNC. Red, Middle (n = 8); Blue, Distal (n = 8). (**b**) Cell proliferation rates were determined using a WST assay. X axis: days; Y axis: OD value (450  nm) (n = 6). Cells in both regions were stimulated with PDGF (25  μg/ml) for seven days and analysed by (**c**) Total collagen assay and (**d**) Sirius Red staining. Open circle, Vehicle (n = 4); Closed circle, PDGF (n = 4). Representative data are shown. (**e**) PDGF-stimulated cells at day 3 were analysed by qRT-PCR. Ligamentous-related genes: COL1, COL3, and TNC; Open circle, Vehicle (n = 4); Closed circle, PDGF (n = 4). (**f**) PDGF-stimulated cells at day 3 were analysed by immunofluorescence. Green, SMA-alexa-488; Red, COL1-cy3; Blue, DAPI; Scale bar: 50 μm. Representative image data are shown (n = 3). Error bars show standard error of the mean. *p < 0.05.

Remnant cells of the middle third region showed significantly higher expression of basal ligamentous differentiation-related genes (e.g., collagen type 1 and tenasin C) than those from the distal third region (p = 0.013 and p = 0.044, respectively) (Fig. 5a). Since Platelet-derived growth factor (PDGF) is known to facilitate ACL graft remodelling and ligamentization, PDGF was given in a dose-dependent manner to assess proliferation and collagen analysis of both middle and distal third regions. PDGF has a growth-increasing effect on the both regions (Fig. 5b). The effect of PDGF on total collagen synthesis of both the middle and distal third regions was reproducible (Fig. 5c,d); however, increased expressions of collagen type 1 (COL1) collagen type 3 (COL3) and tenasin C (TNC) were significantly greater in the middle third region compared to the distal third region in response to PDGF (Fig. 5e). In particular, elevated COL1 expression of distal third region by PDGF was confirmed by immunostaining (Fig. 5f). Therefore, the ligamentous differentiation status with PDGF stimulation was relatively higher in the middle third region than in the distal third region.

## Discussion

This study demonstrated that ACL remnants have different potentials in cell differentiation based on their location. The distal third region of the ACL remnant had a strong tendency for chondrogenic differentiation compared to the middle third region, where a strong tendency for osteogenic and ligamentous differentiation was found.

Peterson *et al*.^19^ investigated the human ACL microstructure and found that the distal region of the ACL, approximately 5–10  mm proximal to the tibial insertion, was predominantly populated with round- to ovoid-shaped cells that resemble chondrocytes, whereas the middle third region was predominantly populated with elongated fibroblasts. In the present study, cells in the ACL distal third were more strongly stained and expressed higher mRNA protein levels for CD34 and chondrogenic differentiation markers (ACAN, collagen 2, and SOX9). This indicates the presence of more blood vessels and a stronger potential for chondrogenic differentiation as compared to cells in the middle third. On the other hand, the more proximal portion of the remnants stained and expressed higher mRNA protein levels for osteogenic (e.g., ALP) and ligamentous differentiation markers (e.g., collagen 1, collagen 3, and TNC). Mifune *et al*.^14^ reported that ruptured ACL remnant tissue included more abundant CD34^+^ vascular-derived stem cells than the non-injured ACL mid substance, which further highlighted the finding that CD34^+^ cells had a higher potential for proliferation and multilineage differentiation. Furthermore, Matsumoto *et al*.^17^ tested for the maturation of bone-tendon integration in a dog model of ACL reconstruction and found endochondral ossification-like integration with enhanced angiogenesis in the grafts of tissue treated with CD34^+^ cells. In the present study, we further divided the location of ruptured ACL remnant tissue and found that CD34^+^ vascular-derived stem cells were more abundant in the distal third portion of the ACL remnant than in the middle third portion of the injured ACL substance. However, characteristics of the stem cell differentiation potential seemed to be more dependent on the location (e.g., cell type) of the remnants, rather than on the abundance of CD34^+^ vascular-derived cells, as the middle third portion of the remnants showed a stronger tendency for osteogenic and ligamentous differentiation even with a smaller amount of CD34^+^ vascular-derived stem cells.

Remnant-preserving ACLR has gained attention due to proprioceptive function and accelerated remnant cell healing potential^10,20–24^. Mechanoreceptors distributed in the ACL and remnant tissue contribute to proprioception of the knee. Although research on mechanoreceptor regeneration and its function in an ACL-reconstructed knee is lacking, surgeons tend to preserve some of the tibial ACL remnants in order to improve knee function. Zhang *et al*.^25^ performed a randomized controlled trial for remnant-preserving ACLR in order to investigate the effect of remnant preservation on tibial tunnel enlargement using a hamstring autograft. The authors found that tibial remnant preservation can resist tibial tunnel enlargement, demonstrating that the remnant tibial side has tendon-bone healing potential. Ahn *et al*.^20^ reported on an ACLR technique using remnant preservation covering the entire graft, and they observed good clinical results with intact ACL grafts in 45 out of 48 reconstructed knees. Similarly, in a retrospective study of 218 patients from Takazawa *et al*.^26^, the authors reported significantly reduced graft rupture rates in the remnant-preservation group, finding only one graft rupture out of 85 knees, demonstrating that remnants covering the entire graft lower the graft rupture rate. There are several studies reporting second-look findings after remnant-preserving technique. Kim *et al*.^27^ assessed 66 consecutive patients who underwent second-look arthroscopy after ACLR using a hamstring autograft and found that cases with 50% or more preserved remnant showed thicker graft tissue with better synovial converage. Guo *et al*.^28^ performed second-look arthroscopy after ACLR using a bone-patellar tendon-bone allograft, and found that patients with no remnant or lower position remanant had poorer synovium coverage. Furthermore, Noh *et al*.^29^ studied the effect of remnant-preservation on achilles allograft, and also found better synovial coverage of the allograft after remnant-preserving and retensioning ACLR. Despite numerous studies, the remnant volume or the extent of remnant preservation necessary for promoting tendon-bone healing or graft maturation is still unclear. In the present study, ACL remnant cells, regardless of their location, showed similarly high expansion and multilineage differentiation potential. However, the distal third region of the ACL remnant showed a stronger tendency for chondrogenic differentiation compared to the middle third region, where a stronger tendency for osteogenic and ligamentous differentiation was found. During remnant-preserving ACLR or when harvesting ACL remnants for tissue engineering purposes, surgeons may consider the characteristics of ACL remnants by their location to predict the healing potential^30^.

The healing potential of the remnant is influenced by a number of different factors, which suggests that the characteristics of individuals should be considered^18,25,31–34^. Nakano *et al*.^31^ found that ACL-derived cells from a younger group enhanced bone-tendon healing in an immunodeficient ACL reconstruction rat model. It has also been reported that ACL remnants in younger patients exhibited higher proliferation and multilineage differentiation potential. This potential decreased with age, as CD34^+^ cells were more prevalent in ACL remnants from younger patients. Naraoka *et al*.^32^ demonstrated a time-dependent alteration in gene expression patterns that decrease over time in ruptured ACL tissue. Moreover, Zhang *et al*.^25^ reported that ruptured ACL remnants extracted during the early phase (within three months) of ACL injury displayed a higher proliferation and multilineage differentiation potential than remnants extracted during the chronic phase. The authors observed that CD34^+^ cells were more prevalent in ACL remnants from an early phase of injury compared with those from the chronic phase. Furthermore, Kirizuki *et al*.^33^ studied ACL healing potential by morphologic pattern (attachment of the remnants to surrounding tissues) and found a significantly higher number of CD34^+^ cells in the non-reattachment group as compared to the reattachment group.

This study has several limitations. First, ACL length differences among individuals were not considered. We uniformly applied a 10-mm to 20-mm mark from the tibial insertion as the distal and middle third boundaries. However, the average total intra-articular ligament length is approximately 32  mm, so it should be reasonable to apply these parameters^19,35^. Second, excised ACL remnants may have been mixed with synovial tissues, which may have influenced the results. However, as we have devoted the best effort to removing synovial tissue macroscopically, this issue should have been minimized. Third, the anterior-posterior axis (bundles) of the ligament was not considered, and this issue may have caused heterogeneity in the tissue samples. Fourth, only ACL ruptures that occurred at the femoral attachment were included for the purpose of the study, and therefore the findings of the study cannot be generalized to all ACL injuries. In addition, although not completely transected, injury may have also occurred on the harvested remnant tissue, which may have influenced the results. However, as remnant-preserving ACLR and tissue engineering using ACL-derived stem cells are based on such injured remnants, results obtained in this study should be noteworthy. Finally, only acute ACL ruptures (within four weeks of injury) were assessed in this study. Therefore, characterization of the remnant cells in chronic or subacute situations remains unknown.

In conclusion, our results demonstrated that ACL remnants have different potentials for cell differentiation based on their location. The distal third region of the ACL remnant showed a stronger tendency for chondrogenic differentiation with higher expression of CD34^+^ cells. On the other hand, the more proximal portion of the remnants had a stronger tendency for osteogenic and ligamentous differentiation. Characteristics of the ACL remnant tissue should be considered when performing remnant-preserving ACLR or harvesting ACL remnants for tissue engineering.

## Materials and Methods

### Patients

This study was carried out in accordance with institutional guidelines and approval from the Ethics Committee of Hanyang University Hospital, with written informed consent from all subjects (IRB-2018-05-001). Between March and June 2018, ruptured ACL remnants were extracted from 11 patients (8 males, 3 females) with a mean age of 23.8 ± 4.69 years (range, 17–31 years), who had undergone primary ACL reconstruction within four weeks following injury. In case of a participant under age of 18 years, we had obtained parental consent. Patients <40 years of age who had a complete ACL rupture of femoral origin with at least 20  mm of intact ligament from the tibial insertion were included in the study. Remnants were extracted en-bloc, marked at the distal third (within 10  mm from the tibial insertion) and middle third (between 10–20  mm from the tibial insertion) and dissected using a number 11 blade. The most distal remnant at the tibial insertional area was preserved to prevent iatrogenic injury to the anterior root of the lateral meniscus. Supplementary table 1 shows the patient demographic data.

### Isolation of human primary ACL remnant cells

The distal and middle thirds of the ACL remnant were each cut into 1-cm or smaller segments with scissors. The segments were enzymatically digested with 1  mg/ml collagenase-type I (Gibco, 17100–017; Sigma, C0130) in serum-free DMEM containing penicillin-streptomycin antibiotics (Thermo Fisher, 15140122) and incubated at 37 °C for 16–20  hours. Cell suspensions were filtered through a nylon mesh, washed in serum-free DMEM several times, and seeded for culture.

### Osteogenic, chondrogenic, and ligamentous differentiation

Osteogenic differentiation methods and activity assessments have been previously reported^36^. Briefly, cells from the distal and middle ACL regions were seeded in growth medium (DMEM with high glucose, Hyclone, SH30243.01) and then differentiated with osteogenic medium (ascorbic acid, dexamethasone, and β-glycerophosphate) into mature osteoblasts. Early stages of differentiation were assessed by ALP staining and activity. Late stages were assessed by quantification of alizarin red S (ARS) staining. For chondrogenic differentiation, cells were pelleted in DMEM/F-12 medium supplemented with infulin-transferrin-selenious acid (ITS) mixture, sodium pyruvate, ascorbate-2-phosphate, dexamethasone, and TGFβ1 in a 15-ml conical tube^37^. Chondrogenic differentiation was assessed by toluidine blue and safranin O staining. For ligamentous differentiation, cells were stimulated with human PDGF-BB (PeproTech, 100-14B) according to the indicated duration and dose^38,39^. The ligamentous status of stimulated cells was assessed by Picro Sirius Red staining (Abcam, ab150681) and total collagen assay (BioVision, K218), according to the manufacturer’s instructions. The capacity of ligamentous differentiation status was defined as collagen synthesis using Picro Sirus Red staining and total collagen assay and COL1, COL3, and TNC expression using qRT-PCR. Differentiation medium was changed every three days.

### Cell proliferation assay

Cell proliferation was assessed using water-soluble tetrazolium salt (WST) (DoGen, EZ-1000), according to the manufacturer’s instructions. Briefly, cells were seeded in 96-well plates at a density of 1E10^3^ cells per well (n = 3) with DMEM growth medium. WST solution was added directly to the cells as indicated, and the cells were then incubated for 1  h to allow the WST to metabolize to formazan. Absorbance was measured using a microplate plate reader at 450  nm.

### qRT-PCR and immunoblotting

RNA and protein extractions were performed as previously described^40^. RNA and proteins were extracted from stimulated cells with NucleoZOL and 1X RIPA buffer, respectively. Complementary DNA was generated from 1  μg of total RNA with reverse transcriptase (Thermo Scientific, EP0442). The cells were lysed with 1X RIPA buffer containing phosphatase (Cell signaling, 5870 S) and protease (Calbiochem, 535140) inhibitors. Proteins were quantified with a Bradford assay. A total of 20–50  μg of protein was subjected to immunoblotting.

The qRT-PCR primers used are as follows: ALP forward, 5′-ACGAGCTGAACAGGAACAACGT-3′; ALP reverse, 5′-CACCAGCAAGAAGAAGCCTTTG-3′; RUNX2 forward, 5′-TGAGCTGAGAGGACATATGGCC-3′; RUNX2 reverse, 5′-TAGACACCAAACTCCACAGCCC-3′; COL1 forward, 5′-AGTGGTTTGGATGGTGCCAA-3′; COL1 reverse, 5′-GCACCATCATTTCCACGAGC-3′; SOX9 forward, 5′-CTGAACGAGAGCGAGAAGCG-3′; SOX9 reverse, 5′-CCCGTTCTTCACCGACTTCC-3′; COL2 forward, 5′-CAACCAGGACCAAAGGGACA-3′; COL2 reverse, 5′-ACCTTTGTCACCACGATCCC-3′; COL3 forward, 5′- CTTCTCTCCAGCCGAGCTTC-3′; COL3 reverse, 5′- CCAGTGTGTTTCGTGCAACC-3′; TNC forward, 5′- GGTTGCTGGAGACTGTGGAA-3′; TNC reverse, 5′- AGGTTTTCCAGAAGGGGCAG-3′; CD34 forward, 5′-CTCCAGCTGTGCGGAGTTTA-3′; CD34 reverse, 5′-TTGGCCAAGACCAGCAGTAG-3′; OCN forward, 5′-ATGAGAGCCCTCACACTCCT-3′; OCN reverse, 5′-CTTGGACACAAAGGCTGCAC-3′; ACAN forward, 5′-TGGGAACCAGCCTATACCCCAG-3′; ACAN reverse, 5′-CAGTTGCAGAAGGGCCTTCTGTAC-3′

The antibodies for immunoblotting were as follows: SOX9 (Merck Millipore, AB5535), ALP (Santa, 365765), RUNX2 (Cell signaling, 12556), β-actin (Cell signaling, 3700), and GAPDH (Cell signaling, 2118).

### Immunofluorescence (IF)

Immunofluorescence was performed as previously described^40^. Briefly, cells were washed with 1X PBS (calcium and magnesium free) and fixed with 4% paraformaldehyde for 10  min at room temperature. Cells were then washed with 1X PBS three times. Cells were permeabilized in PBS containing 0.3% Triton X-100 and 10% BSA for 1  h and then washed three times with PBS. The cells were immunostained with primary antibodies diluted in PBS with 10% BSA at 4°C overnight. Next, cells were washed with 1X PBS for 10  min three times and incubated with CY3- or Alexa Fluor 488-labeled secondary antibodies for 1 h. Nuclei were counterstained with 4, 6-diammidino-2-phenylindole (DAPI) (Vectashield, H1200). Immunofluorescence images were analysed by confocal microscopy (Leica Microsystems, Wetzlar, Germany). The antibodies for immunofluorescence are as follows: COL1A1 (Santa, 8784), SMA (Santa, 53142), SOX9 (Abcam, ab185966), and ALP (Santa, 365765).

### Fluorescence-activated cell sorting (FACS)

Isolated cells were fixed with 2% formalin and stained with CD34-APC (Biolegend, 343607), CD90-PE (Biolegend, 328109), CD105-PE (Biolegend, 323205), CD146-APC (Biolegend, 361015), IgG2a-APC (Biolegend, 400221), or IgG1-PE (Biolegend, 400112) for 10  min at 4°C. Following the staining, the cells were washed with 1X PBS containing 0.5% BSA and 0.1% sodium azide and analysed by flow cytometry (FACS Canto II, BD Biosciences).

### Statistical analysis

Data were generated and analysed with a two-tailed paired t-test using GraphPad Prism 6 software. All data are expressed as mean ± standard deviation from at least three independent experiments.

## Supplementary information

Supplementary Data.

## Data Availability

The data that support the findings of this study are available from the corresponding author upon reasonable request.
