# Supplementary material for: Anterior cruciate ligament remnant cells have different potentials for cell differentiation based on their location
Source: Sci Rep. 2020 Feb 20;10:3097. doi: 10.1038/s41598-020-60047-w (PMC7033160; doi:10.1038/s41598-020-60047-w)
Supplement: Supplementary file 1 — Supplementary Data. [file 41598_2020_60047_MOESM1_ESM.pdf]

Supplementary information

Anterior cruciate ligament remnant cells have different potentials for cell differentiation based on their location

Jin Kyu Lee, Sungsin Jo, Young Lim Lee, Hyosun Park, Jun-Seob Song, Il-Hoon Sung, and Tae-Hwan Kim

Table 1. Patient Characteristics

Patient	Age (yr)	Sex	Time from injury to surgery (wk)	Meniscal treatment	Chondral lesion requiring treatment
1	22	M	2	none	none
2	24	F	3	partial medial menisectomy	none
3	31	M	3	none	none
4	28	M	2	none	none
5	25	M	3	none	none
6	26	M	3	lateral meniscus repair	none
7	17	F	3	partial lateral menisectomy	none
8	24	F	3	none	none
9	18	M	4	none	none
10	18	M	3	none	none
11	29	M	3	none	none

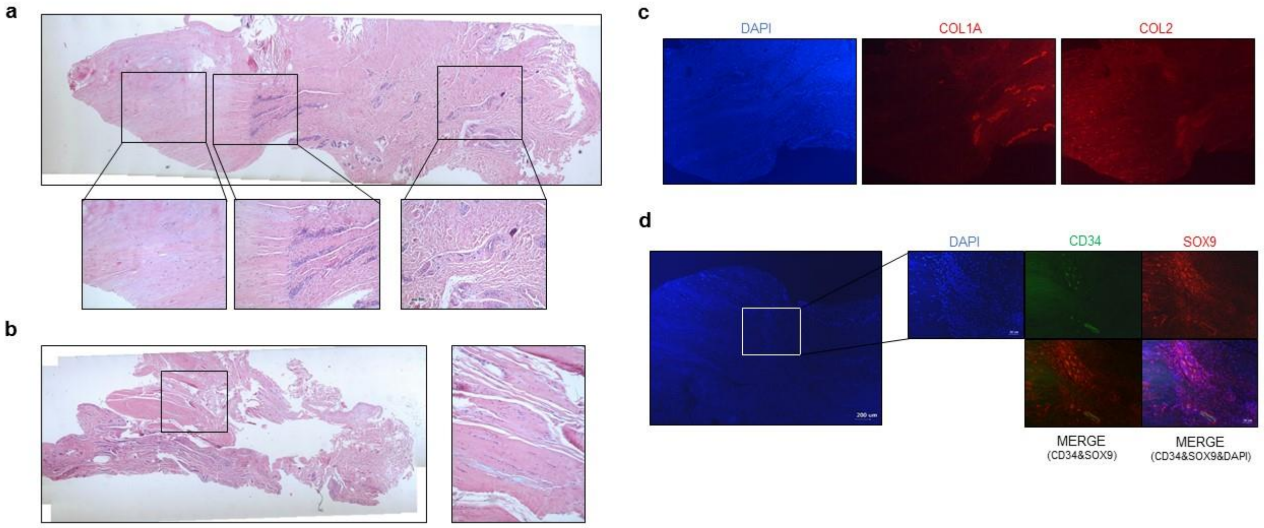

Supplementary figure S1. Tibial insertion site of the anterior cruciate ligament (ACL) and related histological analysis. Histological assessment with hematoxylin/eosin (H&E) staining and microscopic examination of ACL tissue from the (a) distal and (b) middle regions. (c) Immunostaining of collagen type 1 and 2 in the transition zone. (d) Co-expression of CD34 and SOX9 in the transition zone of the ACL were detected by immunostaining. Green, CD34-alexa-488; Red, SOX9-cy3; Blue, DAPI; Scale bar: 200 μm. Representative images are shown.

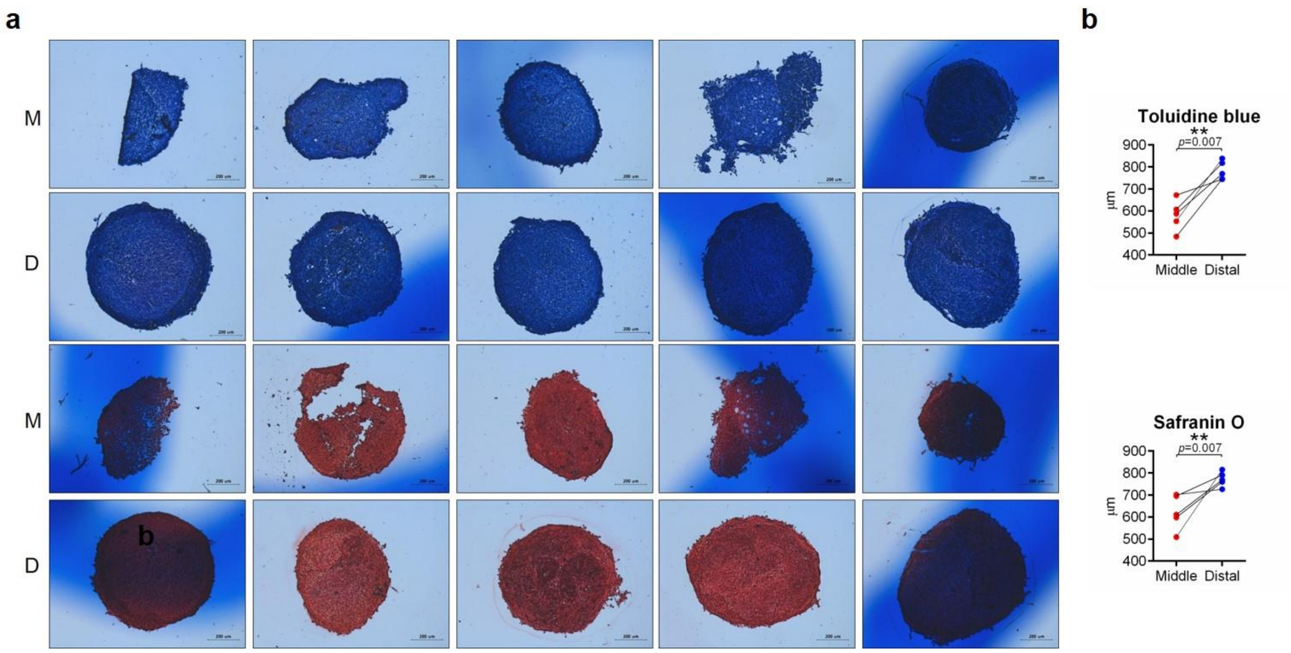

Supplementary figure S2. Comparison of chondrogenic potential in cells from middle and distal third of ACL remnant regions. (a) Both cells were induced into chondrogenic differentiation cryosectioned, and stained with Toludine blue (indicated blue color) and Safranin O (indicated red color). (b) Differentiated cell sizes were assessed. Red, Middle (n=5); Blue, Distal (n=5). This result is microscopy raw images for the quantification of (a). Scale bar: 200 μm. ***p*<0.01.

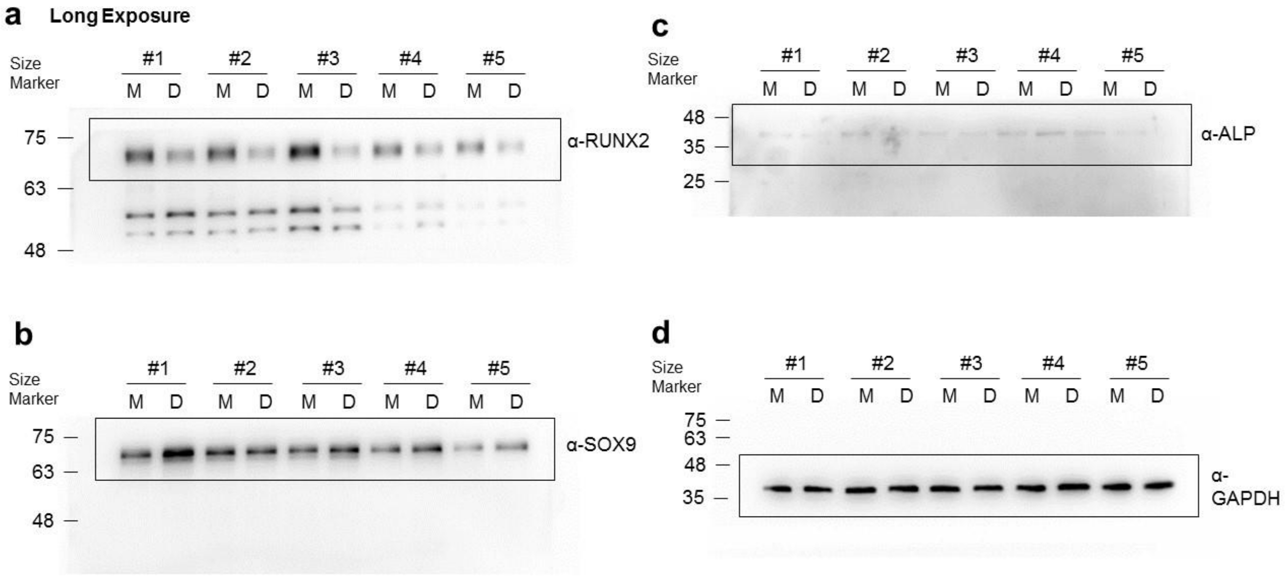

Supplementary figure S3. Uncropped images of the immunoblotting used in Fig. 2d. a, RUNX2; b, SOX9; c, ALP; d, GAPDH.

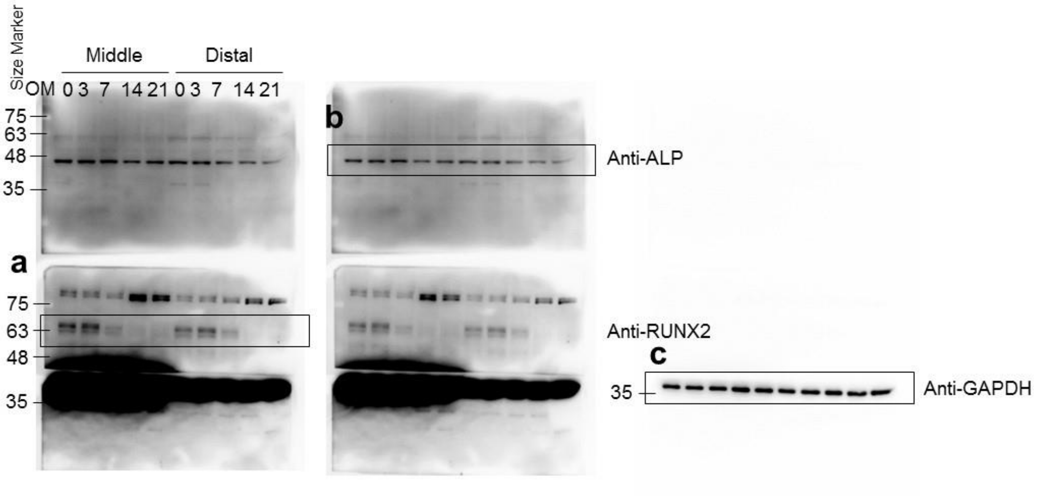

Supplementary figure S4. Uncropped images of the immunoblotting used in Fig. 3c. a, RUNX2; b, ALP; c, GAPDH.
